# Impact of osmotic stress and ethanol inhibition in yeast cells on process oscillation associated with continuous very-high-gravity ethanol fermentation

**DOI:** 10.1186/1754-6834-6-133

**Published:** 2013-09-16

**Authors:** Liang Wang, Xin-Qing Zhao, Chuang Xue, Feng-Wu Bai

**Affiliations:** 1 School of Life Sciences and Biotechnology, Dalian University of Technology, 2 Linggong Rd., Dalian 116023, China; 2 School of Life Sciences and Biotechnology, Shanghai Jiao Tong University, 800 Dongchuan Rd., Shanghai 200240, China

**Keywords:** VHG fermentation, *Saccharomyces cerevisiae*, Process oscillation, Osmotic stress, Ethanol inhibition

## Abstract

**Background:**

VHG fermentation is a promising process engineering strategy aiming at improving ethanol titer, and thus saving energy consumption for ethanol distillation and distillage treatment. However, sustained process oscillation was observed during continuous VHG ethanol fermentation, which significantly affected ethanol fermentation performance of the system.

**Results:**

Sustained process oscillation was investigated in continuous VHG ethanol fermentation, and stresses exerted on yeast cells by osmotic pressure from unfermented sugars and ethanol inhibition developed within the fermentation system were postulated to be major factors triggering this phenomenon. In this article, steady state was established for continuous ethanol fermentation with LG medium containing 120 g/L glucose, and then 160 g/L non-fermentable xylose was supplemented into the LG medium to simulate the osmotic stress on yeast cells under the VHG fermentation condition, but the fermentation process was still at steady state, indicating that the impact of osmotic stress on yeast cells was not the main reason for the process oscillation. However, when 30 g/L ethanol was supplemented into the LG medium to simulate the ethanol inhibition in yeast cells under the VHG fermentation condition, process oscillation was triggered, which was augmented with extended oscillation period and exaggerated oscillation amplitude as ethanol supplementation was increased to 50 g/L, but the process oscillation was gradually attenuated when the ethanol supplementations were stopped, and the steady state was restored. Furthermore, gas stripping was incorporated into the continuous VHG fermentation system to *in situ* remove ethanol produced by *Saccharomyces cerevisiae*, and the process oscillation was also attenuated, but restored after the gas stripping was interrupted.

**Conclusions:**

Experimental results indicated that ethanol inhibition rather than osmotic stress on yeast cells is one of the main factors triggering the process oscillation under the VHG fermentation condition, and in the meantime gas stripping was validated to be an effective strategy for attenuating the process oscillation.

## Background

The budding yeast *Saccharomyces cerevisiae* is the dominant species for ethanol production [1,2]. Compared to batch operation, continuous fermentation can improve productivity to save capital investment on production facilities, and in the meantime save labor and maintenance costs, which has been practiced for large scale production of fuel ethanol in industry. For example, all the four large fuel ethanol plants in China are operated continuously. However, low ethanol concentration in the effluent makes downstream processes such as ethanol distillation and stillage treatment more energy-intensive, particularly when the stillage is treated by the multi-evaporation process that consumes 40-45% of the total thermal energy [3]. To address this issue, VHG fermentation with mash containing total sugars in excess of 250 g/L was developed [4], but unfortunately sustained oscillation was observed with process parameters including sugar, ethanol and biomass concentrations as the operation was extended [5].

Oscillations have been reported with *S. cerevisiae* under different culture and fermentation conditions. Glycolytic oscillation was first observed when a glucose pulse was applied after the system was aerated vigorously [6], but this kind of oscillation was characterized by a short oscillation period less than 1 min, and in the meantime not sustainable and damped gradually. Metabolite assay of yeast cell suspension revealed the crossover point at the enzymatic reaction catalyzed by phosphofructokinase and allosteric regulation of the enzyme, in particular its substrate inhibition by ATP and product activation by AMP and fructose 1,6-bisphosphate [7,8], although contributions by other intermediates downstream the glycolytic pathway such as acetaldehyde and the upstream hexose transport were identified thereafter [9-11], indicating the dynamic nature and distributed control of the major catabolic pathway. For continuous aerobic culture of *S. cerevisiae*, sustained oscillations occurred, and oscillation periods were longer, from minutes to a few hours depending on the medium composition including carbon sources, nitrogen levels and sulphate to produce singling molecules such as acetaldehyde and H_2_S by corresponding pathways as well as culture conditions like pH, dissolved oxygen and dilution rate [12-14]. The underlying mechanisms for the process oscillations were identified to be the synchronization of the population metabolism and cell cycles under specific physiological and culture conditions, due to the asymmetrical budding growth nature of *S. cerevisiae *[15-17].

Compared to these oscillations observed with *S. cerevisiae*, particularly the sustained oscillations under continuous aerobic culture conditions, the process oscillation under continuous VHG fermentation condition was significantly different. For example, the oscillation period was as long as 7–10 days [5], making the process oscillation undetectable with most laboratory research that was maintained only 2–3 days at designated conditions such as the multi-stage continuous VHG fermentation system developed by Bayrock and Ingledew [4]. Although similar oscillations are frequently observed in continuous ethanol fermentation with tanks-in-series systems in industry, this phenomenon has been mistakenly attributed to the fluctuations of process parameters such as mash feeding, temperature, pH and so on, which in fact can be controlled precisely without significant fluctuations.

The process oscillation affects ethanol fermentation performance. On the one hand, stresses on yeast cells such as ethanol inhibition are alleviated periodically, which consequently improves their ethanol productivity. On the other hand, the process oscillation fluctuates downstream processes, particularly ethanol distillation that requires relatively constant ethanol concentration in the fermentation broth. Moreover, more sugars could be discharged with the effluent under oscillatory conditions, and ethanol yield that is calculated based on sugars feeding into fermentation systems without deduction of unfermented sugars in industry would be compromised [5]. Without doubt, identifying major factors triggering this phenomenon is a prerequisite for developing effective strategies to attenuate the process oscillation.

During VHG ethanol fermentation, osmotic effect from unfermented sugars and ethanol inhibition developed within the fermentation system are major stresses exerted on yeast cells, which inevitably affect their growth and ethanol fermentation [18,19]. In this article, their impact on the process oscillation was investigated by supplementing non-fermentable xylose and ethanol into LG medium to simulate the osmotic stress and ethanol inhibition that yeast cells experienced under the VHG fermentation condition. Moreover, gas stripping was incorporated into the VHG fermentation system to in situ remove ethanol produced by *S. cerevisiae* to further study the impact of ethanol inhibition in yeast cells on the process oscillation.

## Results and discussion

### Process oscillation associated with continuous VHG ethanol fermentation

**Figure 1:**
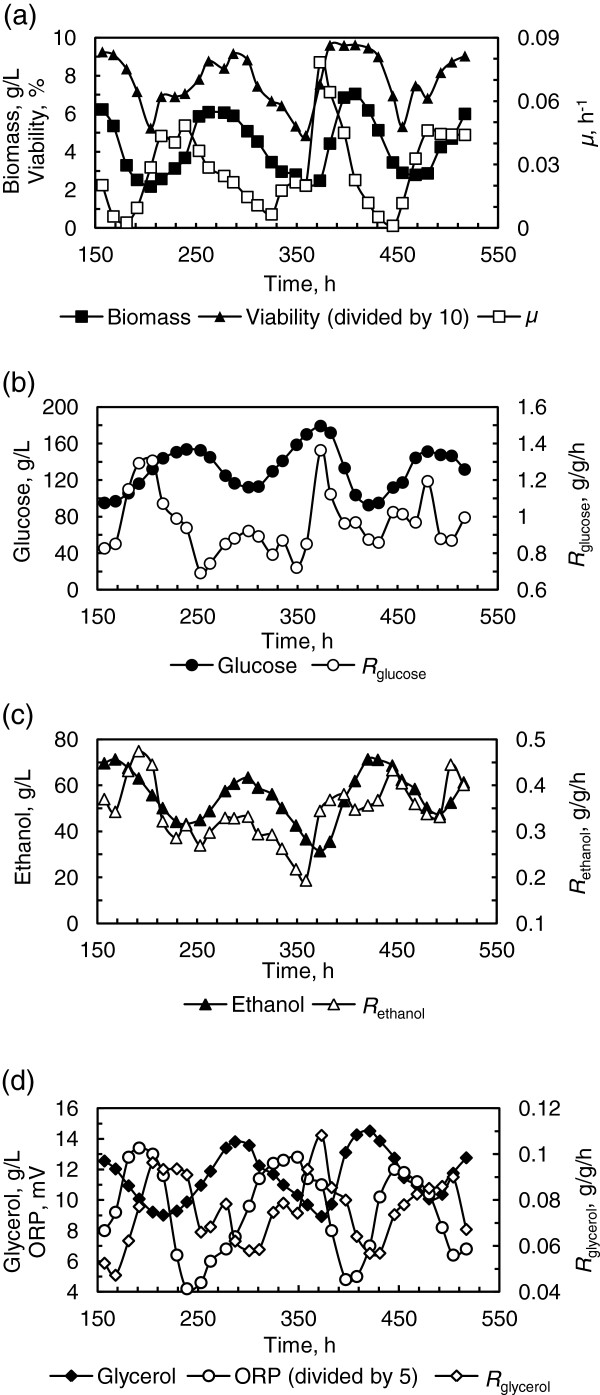
**Sustained oscillation of continuous ethanol fermentation by *S. cerevisiae*.** The VHG medium containing 280 g/L glucose was fed at the dilution rate of 0.027 h^-1^. **(a)** Biomass, specific growth rate (*μ*) and cell viability (divided by 10); **(b)** Residual glucose and specific rate of glucose consumption (*R*_*glucose*_); **(c)** Ethanol and specific rate of ethanol production (*R*_*ethanol*_); **(d)** ORP, glycerol and specific rate of glycerol production (*R*_*glycerol*_).

Previous studies indicated that continuous ethanol fermentation with the LG medium by *S. cerevisiae* was at steady state, but process oscillation developed under VHG ethanol fermentation conditions [5]. Figure 1 illustrates the oscillation profiles recorded for three intact periods from 150 h to 510 h, and oscillation amplitudes, peaks and troughs, and averages of process parameters are summarized and compared with those observed at steady state with the LG medium in Table 1.

**Table 1:** **Fermentation parameters for continuous ethanol fermentations by *S. cerevisiae *** **at steady state with the LG medium and process oscillation with the VHG medium**

**Parameter**	**Steady state with the LG medium**	**Process oscillation with the VHG medium**		
		**Peak/Trough**	**Amplitude**	Average^*^
**Biomass, g(DCW)/L**	11.7	7.1/2.2	4.9	4.3
**Cell viability, %**	98.1	96.2/48.5	47.7	77.8
**Residual glucose, g/L**	0.1	179.7/92.6	87.1	131.7
**Ethanol, g/L**	53.5	71.3/31.4	39.9	55.2
**Glycerol, g/L**	0.05	14.5/8.9	5.6	11.5
**Glucose uptake, g/L/h**	3.24	5.06/2.72	2.39	4.00
**Ethanol productivity, g/L/h**	1.44	1.93/0.85	1.08	1.49
**Ethanol yield**	0.45	0.71/0.17	0.54	0.37
***R***_***glucose***_**, g/g(DCW)/h**	0.28	1.36/0.69	0.67	0.96
***R***_***ethanol***_**, g/g(DCW)/h**	0.12	0.47/0.19	0.28	0.34

^*^ The averages were calculated based on the analytical data measured with 33 samples in Figure 1.

Under the VHG fermentation condition, glucose concentration oscillated between 92.6 and 179.7 g/L, with an average of 131.7 g/L, and correspondingly ethanol concentration oscillated between 71.3 and 31.4 g/L, with an average of 55.2 g/L. Apparently, such a high glucose concentration exerted significant osmotic stress on yeast cells, which was in accordance with more glycerol production, an average of 11.5 g/L *vs* that of only 0.05 g/L produced in continuous ethanol fermentation with the LG medium under steady state, in which all glucose was consumed, and thus no osmotic stress was exerted on yeast cells, since glycerol is synthesized as a compatible solute in yeast to address osmotic stress as well as a strategy for redox balance [20]. As can be seen in Figure 1 (d), the ORP mainly associated with the redox pairs NADH/NAD^+^ and NADPH/NADP^+^ was also oscillated at the range of 49–97 mV, which might be another reason for the increased glycerol production. As for the specific rates of yeast growth, glucose uptake, and ethanol production, they also oscillated, but phase differences were observed when compared with the oscillatory profiles of biomass, glucose and ethanol, indicating the lag responses of yeast metabolism to environmental stresses.

Compared to the oscillatory process observed with the VHG ethanol fermentation, continuous ethanol fermentation with the LG medium was at steady state. The two fermentation systems were operated at the same dilution rate, with almost the same amount of ethanol produced on average, and thus ethanol productivity and glucose uptake did not change significantly under the VHG fermentation condition, but specific rates for ethanol production and glucose uptake were improved drastically, since biomass concentration was much lower, indicating that yeast cells were more productive under oscillatory conditions. However, ethanol yield, the most important techno-economic factor affecting the production cost of fuel ethanol, was lower due to more glycerol production associated with the process oscillation, making it a necessity for oscillation attenuation by developing suitable strategies based on the understanding of the major reasons triggering this phenomenon.

### Impact of osmotic stress on the process oscillation

Continuous ethanol fermentation by *S. cerevisiae* with the LG medium was at steady state, with 53.5 g/L ethanol produced and all glucose consumed. If the same level of ethanol was maintained within the fermentation system, but osmotic stress was exerted on yeast cells, its impact on the process oscillation could be decoupled from ethanol inhibition.

**Figure 2:**
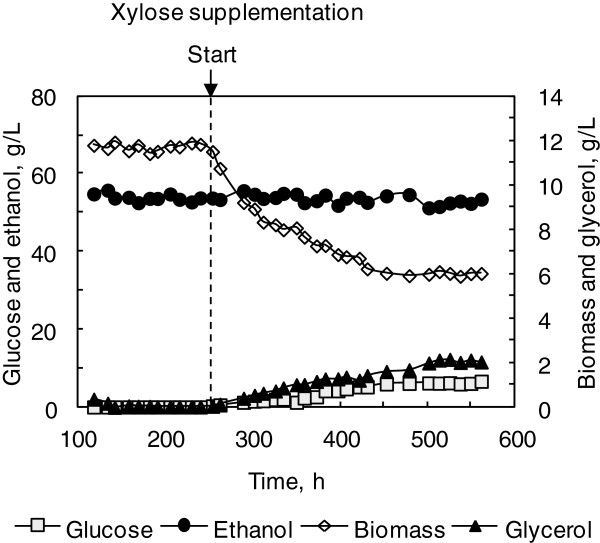
**Impact of osmotic stress on continuous ethanol fermentation by *S. cerevisiae*.** The LG medium containing 120 g/L glucose was fed at the dilution rate of 0.027 h^−1^ to initiate the steady state, and then 160 g/L xylose was supplemented into the LG medium as indicated.

As illustrated in Figure 2, when the LG medium was supplemented with 160 g/L xylose, yeast growth was affected by the osmotic stress, and average biomass concentration decreased to 6.2 g(DCW)/L from 11.7 g(DCW)/L. Consequently, glycerol production increased to 2.2 g/L from 0.05 g/L. Although residual glucose increased slightly to 6.2 g/L from 0.1 g/L, ethanol concentration was 53.0 ± 1.0 g/L, not changed significantly, and the fermentation process was still at steady state, indicating that osmotic stress was not the factor triggering the process oscillation.

Moreover, 2.2 g/L glycerol produced under the osmotic stress condition was substantially lower than that of 11.5 g/L glycerol produced under the oscillatory VHG fermentation condition, indicating that the simulated stressful condition was significantly different from that exerted on yeast cells under the VHG fermentation condition, since glycerol production not only responds to osmotic pressure, but also to redox imbalances and other stressful conditions including ethanol inhibition [21,22]. It is worth noting that the difference between the two fermentation systems was the oscillatory behavior under the VHG fermentation condition, which created variations of osmotic stress and ethanol concentration, and might be the reason for more glycerol production.

### Impact of exogenous ethanol supplementation on the process oscillation

**Figure 3:**
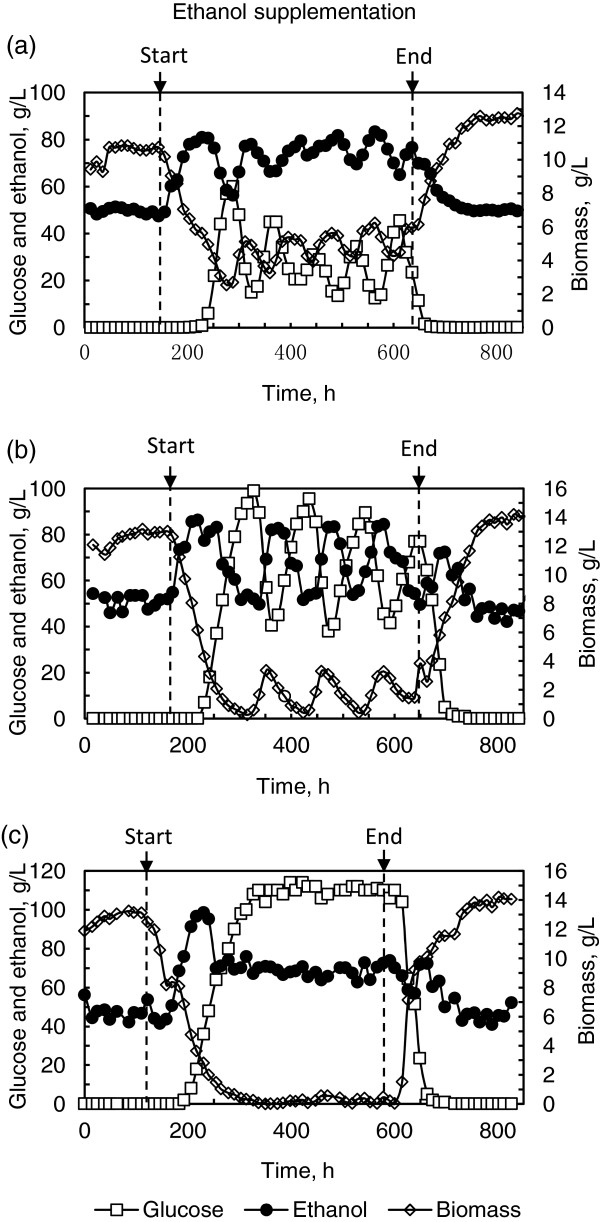
**Impact of exogenous ethanol on continuous ethanol fermentation by *S. cerevisiae*.** Ethanol was supplemented into the LG medium containing 120 g/L glucose at concentrations of 30 g/L **(a)**, 50 g/L **(b)** and 70 g/L **(c)**, respectively. The media were fed into the fermentation system at the same dilution rate of 0.027 h^-1^. Arrows indicate the start and end of the feeding of the ethanol-added medium.

Figure 3 illustrates the impact of ethanol supplementation on the process state. As can be seen, when 30 g/L ethanol was supplemented, biomass, glucose and ethanol concentrations oscillated at a period of about 80 h in the ranges of 3.3-6.2 g(DCW)/L, 12.5-45.1 g/L and 66.4-83.3 g/L, with averages of 4.9 g(DCW)/L, 24.8 g/L and 75.4 g/L. When ethanol supplementation was increased to 50 g/L, biomass, residual glucose and ethanol concentrations still oscillated, but the oscillation period extended to about 105 h, and the oscillation amplitudes exaggerated to 0.4-3.8 g(DCW)/L, 39.0-99.1 g/L and 49.5-84.4 g/L, with averages of 1.9 g(DCW)/L, 67.7 g/L and 66.7 g/L for biomass, glucose and ethanol. Compared to the process state when 30 g/L ethanol was supplemented into the LG medium, biomass accumulation, glucose uptake and ethanol production decreased significantly, indicating that the supplementation of 50 g/L ethanol exerted more severe inhibition in yeast growth and ethanol fermentation, and the extended oscillation period about 105 h was in accordance with the stressful condition exerted on yeast cells. When ethanol supplementation was increased to 70 g/L, no significant process oscillation was observed, and fluctuations of biomass, residual glucose and ethanol concentrations were very small, 0.03-0.6 g(DCW)/L, 106.0-114.1 g/L and 62.8-73.8 g/L, with averages of 0.2 g(DCW)/L, 110.0 g/L and 68.7 g/L, indicating that yeast growth and ethanol fermentation were almost completely inhibited. Since some ethanol was stripped off by air sparged into the fermentor and CO_2_ produced during the fermentation, the trough value of ethanol was slightly lower than that supplemented into the LG medium. When the LG medium without ethanol supplementation was switched back, the process oscillations were attenuated, and the steady state previously observed was restored. These experimental results indicated the role of the ethanol inhibition in the process oscillation.

### Impact of endogenous ethanol on the process oscillation

Followed by continuous ethanol fermentation with the LG medium supplemented with exogenous ethanol, gas stripping was incorporated into the VHG fermentation system to strip off ethanol produced by yeast cells during the fermentation to qualitatively study its impact on the process oscillation. If the process oscillation were attenuated, the impact of ethanol inhibition in yeast cells on the process oscillation under VHG fermentation conditions would be further validated.

**Figure 4:**
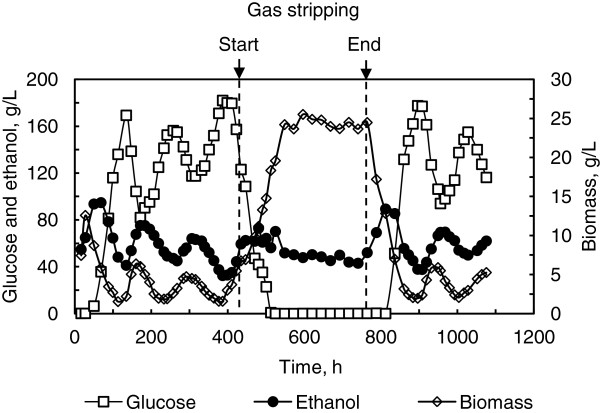
**Impact of gas stripping on continuous ethanol fermentation by *S. cerevisiae*.** The VHG medium containing 280 g/L glucose was fed at the dilution rate of 0.027 h^-1^. Gas stripping with the flow rate of 3.3 L/min was initiated at 435 h and terminated at 765 h.

As can be seen in Figure 4, when the gas stripping was initiated, the process oscillation was gradually attenuated, and quasi-steady state was developed, during which biomass, residual glucose and ethanol concentrations were slightly fluctuated between 23.6-25.5 g(DCW)/L, 0.05-0.10 g/L and 43.9-50.2 g/L, and their averages were 24.3 g(DCW)/L, 0.08 g/L and 48.3 g/L. After the gas stripping was interrupted, the process oscillation was restored. It is worth noting that biomass concentration was increased significantly under the gas stripping condition. Since other volatile by-products stripped off with the gas were negligible due to their low concentrations in the fermentation broth, the main reason for this phenomenon was speculated to be the alleviation of ethanol inhibition in yeast cells, which consequently stimulated their propagation. Although CO_2_ is also inhibitory, this effect is significant only when more CO_2_ is dissolved into the fermentation broth due to the increase of the hydraulic pressure within large fermentors rather than laboratory research with small tanks.

Compared to continuous ethanol fermentation by *S. cerevisiae* from the LG medium with 53.5 g/L ethanol produced at steady state, the average ethanol concentration of 55.2 g/L achieved under the VHG fermentation condition seemed not enough to trigger the process oscillation. Thus, we speculate that ethanol concentration change or ethanol gradient associated with the process oscillation under the continuous VHG fermentation condition and corresponding response of yeast cells to the disruption of intracellular homeostasis would be the underlying mechanism of the process oscillation, which was proposed previously and validated with the oscillatory behavior observed in continuous ethanol fermentation by *Zymomonas mobilis*[23].

### Strategies for process oscillation attenuation

VHG ethanol fermentation was proposed in the early 1990’s [24]. However, almost all studies were carried out at batch fermentations within the past several decades, except one report from Prof. WM Ingledew’s group, the pioneer in VHG fermentation technologies, in which a cascade fermentation system composed of 5 fermentors was employed [4]. Although as high as 16.7% (v/v) ethanol was produced, the dilution rate of the fermentation system was extremely low, about 0.0086 h^−1^, making it not practical in industry. Moreover, only 3 days were maintained at each dilution rate applied to the fermentation system, which might be too short to detect any oscillations.

Process oscillations have been reported in continuous ethanol fermentations by *Z. mobilis* and *S. cerevisiae *[23,25], and corresponding attenuation strategies have been developed. For example, when tubular bioreactors packed with Intalox ceramic saddles were arranged in cascade, following the tank fermentor, the process oscillation observed with continuous ethanol fermentation by *S. cerevisiae* was attenuated, and a quasi-steady state was established [26]. In this study, gas stripping was applied to mitigate the process oscillation for the first time by removing ethanol produced during the fermentation, and quasi-steady state was achieved. Compared with previous studies, residual glucose was maintained below 0.1 g/L under the gas stripping condition, and thus complete glucose conversion was achieved, indicating that it was more effective in enhancing ethanol fermentation under the VHG fermentation condition. On the other hand, ethanol concentration in the condensate collected was as high as 189.0 g/L, which was more suitable for distillation with less energy consumed than other attenuation strategies. Moreover, off-gas produced during ethanol fermentation instead of N_2_ gas could be recycled to strip off ethanol, and no significant difference was observed in the VHG fermentation system. Therefore, gas stripping seems more practical from the viewpoint of industrial application.

## Conclusions

By supplementing non-fermentable xylose and ethanol into the LG medium, ethanol inhibition rather than osmotic stress was validated to be one of the main factors trigging the process oscillation under the VHG fermentation condition. Meanwhile, the process oscillation was effectively attenuated when gas stripping was incorporated into the continuous VHG ethanol fermentation system to in situ remove ethanol produced by yeast cells, which not only further validated the impact of ethanol inhibition in yeast cells on the fermentation process, but also provides an effective strategy for its attenuation.

### Future research

The experimental results validated the hypothesis that ethanol produced during fermentation and its inhibition in yeast cells rather than osmotic stress exerted by glucose remained within the system is one of the main reasons for triggering the process oscillation, which provides the foundation for exploring the gene expression and intracellular metabolism of yeast cells under oscillatory conditions. Based on the work, we are now investigating the transcriptomics and metabolomics profiles of yeast cells under oscillatory conditions associated with continuous VHG ethanol fermentation, with an objective to understand the molecular mechanism underlying this phenomenon. Meanwhile, off-gas strapping could attunate the process oscillation, and improve the productivity of the VHG fermentation system, but detailed energy and mass balance as well as economic analysis need to be performed.

## Methods

### Strain and media

An industrial yeast strain *S. cerevisiae* 4126 provided by Jana Otrubo (Department of Chemical Engineering, the University of Waterloo, Canada) was used in this study. Pre-culture was grown in 500-mL Erlenmeyer flask containing 150 mL medium at 30°C and 150 rpm for 20 h to the middle of the exponential growth phase. The medium for the seed culture composed of 30 g/L glucose, 5 g/L yeast extract and 3 g/L peptone. Another two media were developed for continuous ethanol fermentation: LG medium composed of 120 g/L glucose, 5 g/L yeast extract and 3 g/L peptone, and VHG medium composed of 280 g/L glucose, 5 g/L yeast extract and 3 g/L peptone.

The media for seed culture and LG fermentation were sterilized at 121°C for 20 min, while the VHG medium was sterilized at 110°C for 20 min, and then immediately cooled down to room temperature to avoid side reactions which not only degraded glucose, but also generated inhibitors affecting yeast growth and ethanol fermentation.

### Continuous ethanol fermentation

**Figure 5:**
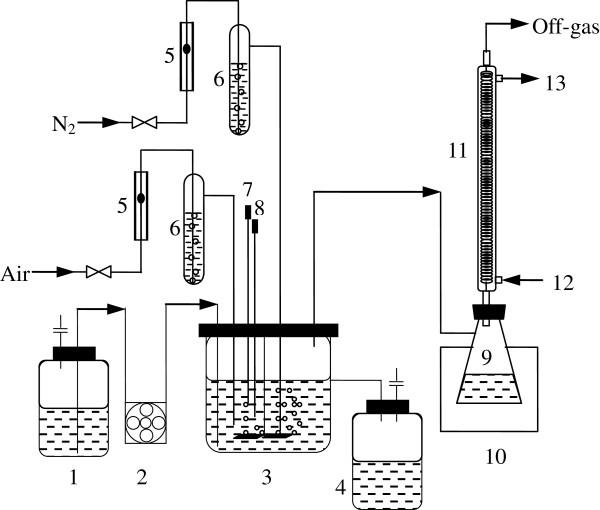
**Schematic diagram of continuous VHG ethanol fermentation with gas stripping.** 1, medium storage tank; 2, peristaltic pump; 3, stirred tank fermentor; 4, fermentation broth storage tank; 5, gas flow meters; 6, humidifiers; 7, pH controlling unit; 8, temperature controlling unit; 9, condensate storage tank; 10, cooling system; 11, condenser; 12 and 13, thermostat cooling water inlet and outlet.

After inoculated with 150 mL seed culture, batch culture was initiated for yeast propagation in a 2.5-L stirred tank bioreactor (KBT-2.5 L, Korea), which contained 1700 mL LG medium, and was operated at 30°C, 300 rpm, 0.05 vvm, and pH 4.50 controlled automatically by adding 2 M NaOH. Continuous ethanol fermentation was started by feeding the LG or VHG medium into the fermentor at the dilution rate of 0.027 h^-1^, and steady state was observed with the LG fermentation, but process oscillation was developed under the VHG fermentation condition [5]. Figure 5 is the process diagram.

Previous studies indicated that the maximal glucose concentration at its oscillation peak was about 180 g/L [5], which exerted an osmotic pressure of 24.9 atm [27]. When 160 g/L xylose was supplemented into the LG medium, it generated an osmotic pressure of 26.5 atm, making the xylose supplementation well simulated the osmotic stress under the VHG fermentation condition. On the other hand, ethanol concentration oscillated with peak values of 70–80 g/L under the VHG fermentation condition [5]. Therefore, ethanol was supplemented into the LG medium at 30 g/L to make the total ethanol concentration at this level. Moreover, higher ethanol concentrations of 50 g/L and 70 g/L were also supplemented into the LG medium to further explore the impact of ethanol inhibition in yeast cells on the process oscillation. All these media were fed into the fermentation system at the same dilution rate of 0.027 h^−1^ as that applied to the continuous VHG fermentation. In addition, gas stripping by N_2_ gas or off-gas produced during ethanol fermentation was incorporated into the system to in-situ remove ethanol produced by *S. cerevisiae* under the VHG fermentation condition and investigate the impact of endogenous ethanol inhibition in yeast cells on the process oscillation, which was performed by sparging the stripping gas into the fermentation system at a flowrate of 3.3 L/min.

### Calculations

The specific rates (h^−1^) of yeast growth, glucose consumption, and ethanol production and major byproduct glycerol *μ, R*_*glucose*_, *R*_*ethanol*_ and *R*_*glycerol*_ were calculated with the following equations:

(1) $$\mu =\frac{1}{X}\cdot \frac{\mathrm{dX}}{\mathrm{dt}}+D$$

(2) $$R_{\text{glucose}}=\frac{1}{X}\cdot \left(D\cdot C_{\text{glucose}0}-D\cdot C_{\text{glucose}}-\frac{dC_{\text{glucose}}}{\mathrm{dt}}\right)$$

(3) $$R_{\text{ethanol}}=\frac{1}{X}\cdot \left(\frac{dC_{\text{ethanol}}}{\mathrm{dt}}+D\cdot C_{\text{ethanol}}\right)$$

(4) $$R_{\text{glycerol}}=\frac{1}{X}\cdot \left(\frac{dC_{\text{glycerol}}}{\mathrm{dt}}+D\cdot C_{\text{glycerol}}\right)$$

Where *D* and *t* represent dilution rate and fermentation time, h^−1^ and h; *C*_*glucose0*_ represents glucose concentration in the medium, g/L; *X*, *C*_*glucose*_, *C*_*ethanol*_ and *C*_*glycerol*_ represent concentrations of biomass, glucose, ethanol and glycerol in the fermentation broth, g/L.

The osmotic stress was calculated by the following equation:

(5) π=iMRT

where

*π*, the osmotic stress in atm

*i*, van 't Hoff factor of the solution

*M*, molar concentration in mol/L

*R* = 0.08206 L · atm/mol · K, universal gas constant

*T*, absolute temperature in K

### Analytical methods

Triplicate samples were taken and centrifuged at 10, 000× *g* at ambient temperature for 5 min to remove yeast cells. The supernatant was stored at −20°C for further analysis. The Waters HPLC system with RI-detector (Waters 410, Waters, MA, USA) was used to analyze glucose, ethanol and glycerol in the effluent. An Aminex HPX 87-H column (300 × 7.8 mm, Bio-Rad Laboratories, Hercules, CA, USA) was used for separating these components. The column was eluted with 5 mM H_2_SO_4_ at a flow rate of 0.5 mL/min with a column temperature of 50°C.

The analytical errors for glucose, ethanol and glycerol were 1.5%, 1.7% and 1.3%, respectively. Analysis of biomass concentration and cell viability were described in the reference [28], and the analytical errors for biomass and cell viability was 2.1% and 6.5%, respectively. All data reported in this work were averages of analysis of the triplicate samples.

## Abbreviations

VHG: Very high gravity; LG: Low gravity; ORP: Oxidation reduction potential; DCW: Dry cell weight.

## Competing interests

The authors declare that they have no competing interests.

## Authors’ contributions

LW, under the supervision of FWB, initiated the research scheme, carried out the experimental work and developed the draft. FWB, XQZ and CX were involved in data interpretation, discussion, and manuscript revision. All authors agreed and approved the submission.
